# Swimmers’ Effective Actions during the Backstroke Start Technique

**DOI:** 10.3390/s23187723

**Published:** 2023-09-07

**Authors:** Karla de Jesus, Kelly de Jesus, Luís Mourão, Hélio Roesler, Ricardo J. Fernandes, Mário A. P. Vaz, João Paulo Vilas-Boas, Leandro J. Machado

**Affiliations:** 1 Centre of Research, Education, Innovation and Intervention in Sport, Faculty of Sport, University of Porto, 4200-450 Porto, Portugal; kellydejesus@ufam.edu.br (K.d.J.); lmourao@eu.ipp.pt (L.M.); helio.roesler@udesc.br (H.R.); ricfer@fade.up.pt (R.J.F.); jpvb@fade.up.pt (J.P.V.-B.); lmachado@fade.up.pt (L.J.M.); 2 Human Studies Development Laboratory, Faculty of Physical Education and Physiotherapy, Federal University of Amazonas, Manaus 69077-000, AM, Brazil; 3 Polytechnic Institute of Porto School of Engineering, 4249-015 Porto, Portugal; 4 Aquatic Biomechanics Research Laboratory, Health and Sports Science Centre, University of the State of Santa Catarina, Florianópolis 88080-350, SC, Brazil; 5 Porto Biomechanics Laboratory, University of Porto, 4200-450 Porto, Portugal; gmavaz@fe.up.pt; 6 Institute of Mechanical Engineering and Industrial Management, Faculty of Engineering, University of Porto, 4200-465 Porto, Portugal

**Keywords:** backstroke, starts, passive force, propulsion

## Abstract

The analysis of the external forces of swimming starts has revealed how swimmers propel themselves out of the block, but data should be properly interpreted to fully understand force-generation mechanisms. This study aimed to assess horizontal and vertical forces in the backstroke start based on swimmers’ structural and propulsive actions. Firstly, a simulated structural force was estimated by two transient backstroke-start inter-segmental realistic body positions: a maximally tucked position and an extended one (just before the hands-off and the take-off, respectively). Secondly, 10 competitive backstroke swimmers performed four 15 m maximal backstroke starts with the external forces estimated. Thirdly, the simulated structural force was subtracted from raw horizontal and vertical force data, measured between hands-off and take-off instants, resulting in the propulsive forces. The application of the algorithm has evidenced that backstrokers’ horizontal and vertical simulated-structural-force components contributed to ~40% of total force during start propulsion (~0.2–0.12 s before the take-off), followed by the propulsive horizontal force increment and a progressive vertical component reduction (~0.05 s) with ~20° take-off angle. Based on these findings, researchers and coaches can better guide swimmers as to the proper mechanical strategies to achieve effectiveness in the backstroke start, and to improve direct transfer of resistance training programs.

## 1. Introduction

The starting time, usually assessed from the beep until the swimmer’s vertex passes the 15 m mark, is highly representative of overall performance for short-distance events [1,2,3]. The start is composed of the block/wall, flight, entry, and underwater interdependent phases [1,2,4], with the first actions being determinant of the global effectiveness [2,3,5]. In the ventral events starts, it is advised to generate proper partition between horizontal and vertical forces to guarantee optimal take-off angles for a greater flight distance [1,5,6]. Flight distance is also important in the dorsal events starts [7,8,9], allowing the swimmers to travel considerably faster through the air than in water. The backstroke start flight distance is shorter compared to that observed on ventral events starts [1,4,10], since the swimmers’ initial position is very close to the water surface [7,9,11]. Moreover, its reduced take-off angle implies a smaller vertical reaction force, which highlights the importance of the horizontal component for propulsion [10,12] and overall performance.

**Figure 1:**
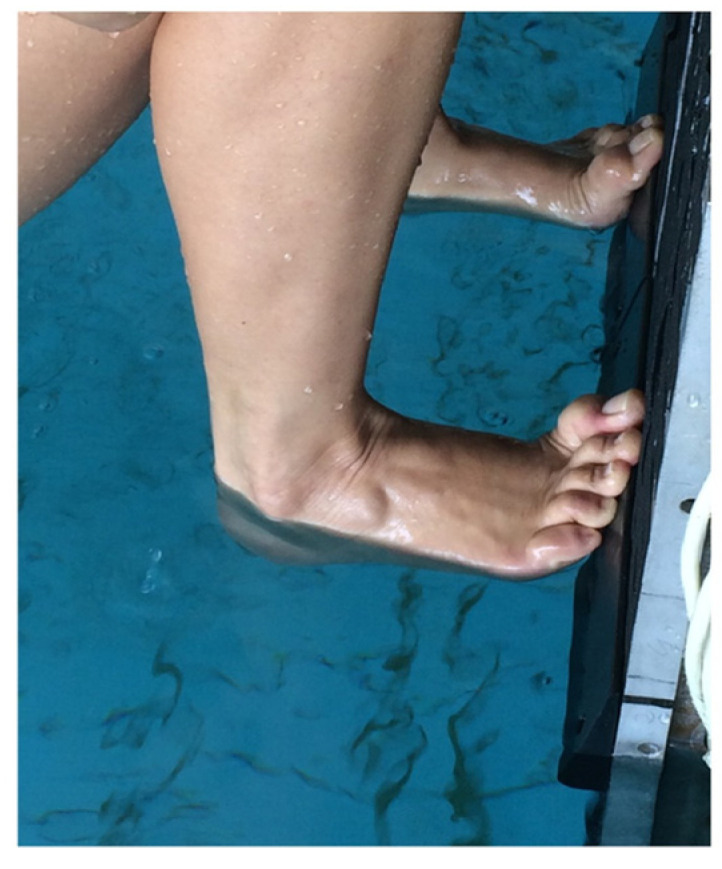
Positioning of a swimmer’s feet on the wedge pair during the wall-contact phase of the backstroke start.

Detailed assessments of the backstroke-start kinetics using tri-axial waterproof force plates are scarce [13], which limits the proposal of effective steering strategies. Previous studies that focused on differences in the lower limbs’ force production between backstroke start technical variants (with and without the wedge) revealed a two-peak profile in the horizontal and vertical raw force components [8,10,13], with no wedge effect on impulse being noticed [8]. Thus, a thorough description of force generation considering postural and effective force components is needed for accurate training feedback (as previously evidenced with the “grab start” technique [6]). The horizontal and vertical force components in the backstroke start might involve swimmers’ muscular and body weight dynamic effects to maintain contact with the wall while moving from the hands-off to the take-off. The use of the wedge might partially obviate the friction mechanism that occurs, especially on the vertical force component [8,10,12], as to swimmers’ feet indentation and wall contact stability (Figure 1).

According to established stepwise procedures [5], the impulse is continuously generated in the absence of an effective start effort. This can be evidenced by an inactive rigid body fall while rotating around a wall-fixed axis. In the backstroke start, the unanimated body’s descent would begin at ~40° to the horizontal axis [11] (similarly to the kick start [1,14,15], and conversely to the ~90° observed in the grab start [6]), which might imply a delayed loss of wall contact given the smaller take-off angle. For an inactive body, the ground reaction force $\left(\vec{GRF}\left(t\right)\right)$ is simply a passive force applied to the swimmers’ inertial structure (${\vec{R}}_{\mathrm{Passive}}\left(t\right)$) (Equation (1)). ${\vec{R}}_{\mathrm{Passive}}\left(t\right)$ should be considered in the ongoing study as the force generated by an inert rigid body fall, for which the calculation of inertia matrix and differential equation solution for force–time curves might be determined [6].

(1) $$\vec{GRF}\left(t\right)={\vec{R}}_{\mathrm{Passive}}\left(t\right)$$

Therefore, it is recommended to split the swimmers $\vec{GRF}\left(t\right)$ into passive (${\vec{R}}_{\mathrm{Passive}}\left(t\right)$) and active (${\vec{R}}_{\mathrm{Active}}\left(t\right)$) backstroke-start force components (Equation (2)) for swimmers’ effective technical instruction [11,16].

(2) $$\vec{GRF}\left(t\right)={\vec{R}}_{\mathrm{Passive}}\left(t\right)+{\vec{R}}_{\mathrm{Active}}\left(t\right)$$

Here ${\vec{R}}_{\mathrm{Passive}}\left(t\right)$ is the same as in Equation (1), and ${\vec{R}}_{\mathrm{Active}}\left(t\right)$ is in the opposite direction of the propulsive force vector applied to the start wall by swimmers’ muscular and mechanical actions.

The current study aimed to apply the $\vec{GRF}\left(t\right)$ splitting formalism [6] to the backstroke start technique after the hands-off instant, since, from this point onwards, a swimmers’ body rotates around a single axis according to the simple rigid body model [6]. When performing the backstroke start, swimmers have the option of using the wedge and the wall (FINA rule SW 6.1; FINA [17]) by means of a halluces–platform alignment, the central point of which should be the centre of pressure (COP). This geometrical profile may be described as the centre of mass (CM) rotation around the halluces lateral–medial axis, merged into the CM displacement along the anterior–posterior CM–COP direction [6]. $\vec{GRF}\left(t\right)$ will be split into the ${\vec{R}}_{\mathrm{Passive}}\left(t\right)$ and ${\vec{R}}_{\mathrm{Active}}\left(t\right)$ components using the algorithm previously proposed [6] to better understand the mechanical load applied by swimmers’ musculoskeletal systems and to define suitable steering strategies during the off-of-the-wall propulsion.

## 2. Materials and Methods

### 2.1. Swimmers’ Matrix of Inertia Determination

**Figure 2:**
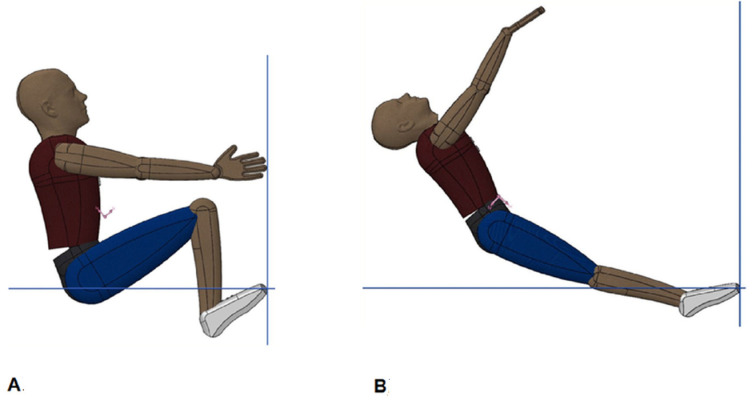
Rigid, articulated body positions mimicking two limit-case transient backstroke-start body positions: the most-tucked and the most-extended ((**A**,**B**) panels). The continuous and dotted blue lines are representative of the model coordinate plane system and the water line.

The minimum and maximum values of the moment of inertia around the halluces (3D CAD, DS Solidworks, Dassault Systèmes, Solidworks Corporation, Waltham, MA, USA) and the segmental values (NASA human body anthropometrical inertial model [18]) were assessed using a swimmer’s model (with sagittal symmetry being assumed) [5]. The swimmer’s matrix of inertia and the estimation of CM–COP distance were necessary for the application of the differential equations [6] governing the rigid body’s fall as a rotation around halluces. These values were assessed with a model of a rigid articulated human body (mass 85.71 kg, volume 89.4 dm^3^ and surface area 3.26 m^2^) compatible with two transient swimmers’ inter-segmental backstroke-start body positions (one maximally tucked and the other maximally extended, with 0.668 m and 1.159 m CM–COP distance just before hands-off and take-off, respectively; Figure 2A,B). The swimmer’s mass had a small effect on the dynamic equations governing the dummy model’s motion [6]. The backstroke start variant with the feet positioned over the wedge (0.04 m above water level) [13] was performed with the hands grasping the grips vertically [10,12].

### 2.2. Experimental Start Protocol

**Figure 3:**
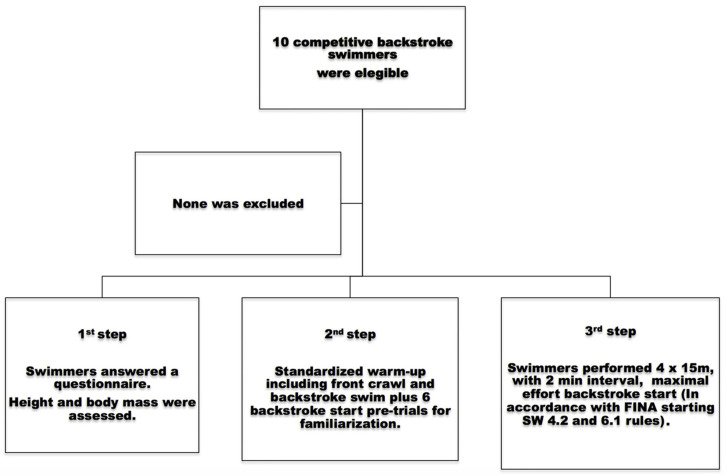
Flowchart of sample recruitment and inclusion process, as well as the experimental steps [19].

**Figure 4:**
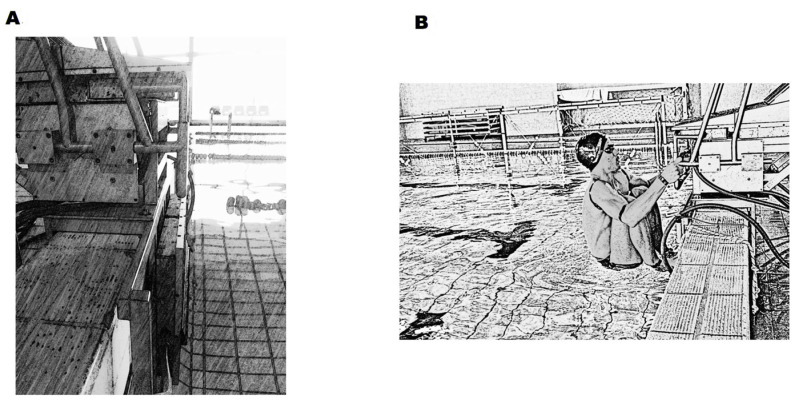
Schematic representation of experimental procedures. Instrumented starting block ((**A**) panel), and the backstroke start with the feet positioned over the wedge (0.04 m above water level), performed with the hands grasping the grips vertically ((**B**) panel).

Ten male competitive backstroke swimmers (age 21.1 ± 5.36 years old, stature 1.78 ± 0.04 m, body mass 72.82 ± 10.05 kg, training background 12.6 ± 6.13 years, 100 m short course backstroke performance 59.67 ± 2.89 s representing 77.69 ± 3.59% of the 2021 world record) volunteered to participate. All swimmers were healthy (with no serious injuries or illnesses in the last six months), able-bodied, and national-level event participants. Approval for all experimental procedures was granted by the local university Ethics Committee in accordance with the Declaration of Helsinki. Swimmers and parents/legal guardians (when swimmers were aged below 18 years) freely provided written informed consent before data collection. A power analysis was performed (G*Power version 3.1, Department of Psychology, Heinrich Heine Universität, Duesseldorf, Germany), and ten participants were required to have an 80% power level to detect an effect size of >0.5 using a one-tailed single-sample t-test with a probability of 0.05. The sample recruitment and the experimental procedures are presented in Figure 3 and Figure 4, respectively.

### 2.3. Data Collection and Analysis

The instrumented start block consisted of four tri-axial strain gauge waterproof force plates, two for the upper limbs and two for the lower limbs, which allowed independent $\vec{GRF}\left(t\right)$ measurements [12]. Upper-limb force plates were laterally fixed on the starting block, with independent handgrips fixed on each force-plate top. Lower-limb force plates were vertically positioned on a custom-built underwater structure fixed on the wall, each one with its independent wedge attached over the force-plate top (at 0.04 m above the water level, cf. FINA Rule FR 2.2.11 [19]). The two force-plate pairs had a 0.5 N sensitivity, <5% error and a 300 and 200 Hz resonance frequency [12], respectively. The start block was fixed over the underwater structure to ensure that the overall dynamometric unit complied with FINA Rules FR 2.1.8 [19]).

The dynamic calibration was made, as described before, on the rigid-body fall [6], which revealed a resultant homogeneity on the static calibration. Custom-designed data processing software was created in LabView 2013 (SP1, National Instruments Corporation, Austin, TX, USA) to acquire, plot, and save the strain readings from each force plate at a 2000 Hz sampling rate. A starter device (Omega StartTime IV Acoustic Start, Swiss Timing Ltd., Corgémont, Switzerland) was instrumented to simultaneously generate a starting command and export a trigger signal to the force plates through a custom-built trigger box (cf. FINA Rule SW 4.2 [17]). $\vec{GRF}\left(t\right)$ records were analogue-to-digital converted by several modules for strain-signals reading (NI9237, National Instruments Corporation, USA) and their respective chassis (CompactDAQ USB-9172 and Ethernet-9188 National Instruments Corporation, Austin, TX, USA).

Two custom-designed processing routines created in Matlab (The MathWorks Incorporated, Natick, MA, USA) converted strain readings (µɛ) into force values (N), filtered upper and lower limb force curves (4th order zero-phase digital Butterworth low-pass filter with a 10 Hz cut-off frequency [10]), summed right- and left-limb force data, and normalized upper and lower limb force values to swimmers’ body weights. The splitting tool algorithm [6] was applied to each of the four trials of the swimmer $\vec{GRF}\left(t\right)$ and was synchronized to the take-off. $GRF_{H}\left(t\right)$ and $GRF_{V}\left(t\right)$ were used to calculate the $\vec{GRF}\left(t\right)$ angle to the horizontal, which, for the rotation around the halluces, is the same as that between the line CM–COP and the horizontal (Equation (3)).

(3) $$\theta \left(t\right)=\arctan\left[GRF_{V}\left(t\right)/GRF_{H}\left(t\right)\right]$$

**Figure 5:**
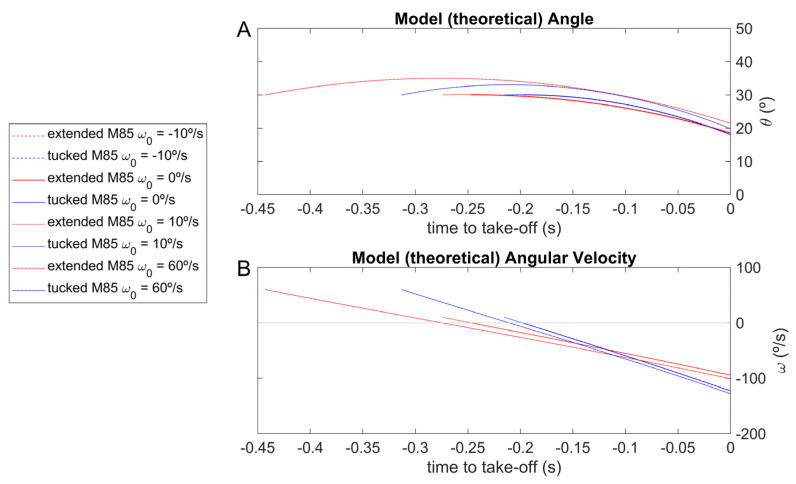
Simulation of the most-extended and the most-tucked rigid body falling at −10°/s (red and blue dashed lines), 0°/s (red and blue continuous lines), 10°/s (red and blue dotted lines), and 60°/s angular velocity (red and blue dot-dashed lines); CM–COP line and, respectively, horizontal axis θ angle and angular velocity ((**A**,**B**) panels), with time synchronized to the take-off.

**Figure 6:**
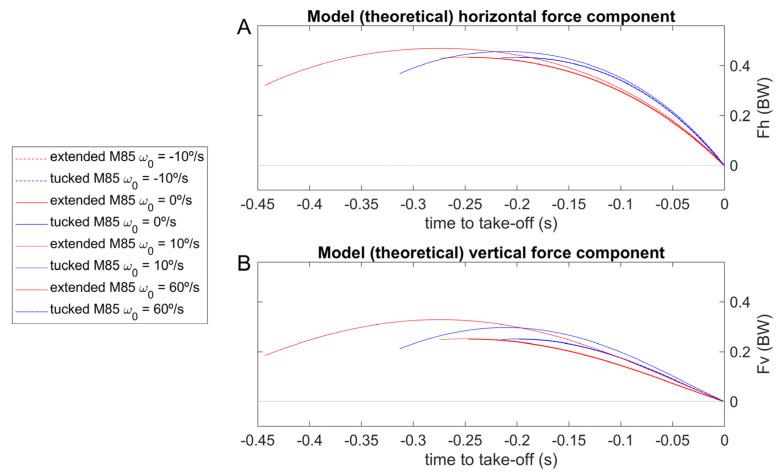
Simulation of the most-extended and the most-tucked rigid body falling at −10°/s (red and blue dashed lines), 0°/s (red and blue continuous lines), 10°/s (red and blue dotted lines), and 60°/s angular velocity (red and blue dot-dashed lines). $GRF_{H}\left(t\right)$ (Fh) and $GRF_{V}\left(t\right)$ (Fv) data ((**A**) and (**B**) panels, respectively) are presented as a fraction of model body weight and synchronized to the take-off.

To compute ${\vec{R}}_{\mathrm{Passive}}\left(t\right)$, the moments of inertia of the rotation around the halluces COP from a rigid body fall were estimated (in the most-tucked and most-extended backstroke start positions; Figure 2, A and B panels). It was necessary to give information about the initial angular velocity and establish the −10, 0, 10 and 60°/s values for the rigid-body models (Figure 2) to run the dynamic model to obtain the angle between the CM–COP line and the horizontal axis, the respective angular velocity, and the horizontal and vertical force components (Figure 5 and Figure 6). The differences between the values returned for simulated CM–COP angle to horizontal axis $\left[\theta \left(t\right)\right]$, respective angular velocity (Figure 5) and $R_{\mathrm{Hpass}}\left(t\right)$ and $R_{\mathrm{Vpass}}\left(t\right)$ theoretical profiles (Figure 6) were small in the simulations and mainly dependent on the body position, and did not led to a clear initial-condition choice. Therefore, the 0°/s initial condition at the hands-off was selected, since the angle is a decreasing function of time. The cases with positive initial angular velocities display the angle as an inverted U-shape, preventing a unique inversion of the angle-to-time curve [6].

## 3. Results

**Figure 7:**
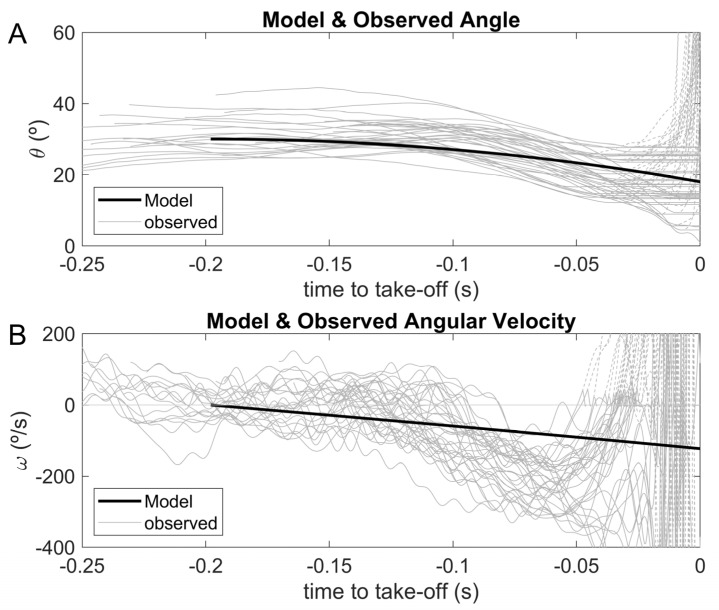
CM–COP line and horizontal axis angle ((**A**) panel) for a 0°/s initial condition, as well as respective angular velocity ((**B**) panel) from the passive rigid-body fall (continuous black line) and the 40 observed swimmers’ backstroke-start trials (continuous grey lines). Model and observed angle and angular velocity were synchronized to take-off.

Complete inertia matrix components were calculated (Table 1) and the moment of inertia around the halluces’$\;(I_{ZZ})$ minimum and maximum values were obtained in the most-tucked and most-extended body geometries, respectively. In both body geometries, $I_{ZZ}\gg I_{YZ},\;I_{XZ}$ justifies the negative meaningfulness of the difference in $I_{YZ}$ and $I_{XZ}$ values between rigid articulated body positions. The value for the inertia term $I_{ZZ}$ almost tripled from the most-tucked to the most-extended inter-segmental backstroke-start positions. The CM–COP line’s angle to the horizontal axis computed during the unanimated rigid body fall showed an ~20° take-off angle (Figure 7A), which is compatible with the trend found in the swimmers’ values. Angular velocity during the unanimated rigid body fall displayed a linear descendent profile from hands-off until take-off, contrary to the observed swimmers’ angular velocity–time curves, which highlighted a steep negative increment in values between ~−0.15 and −0.05 s (Figure 7B). Swimmers’ angle and angular velocity (Figure 7) showed an abrupt positive increase immediately before take-off, explained by near-zero force values, due to platform strain recovery inertial effects, causing numerical instability.

**Figure 8:**
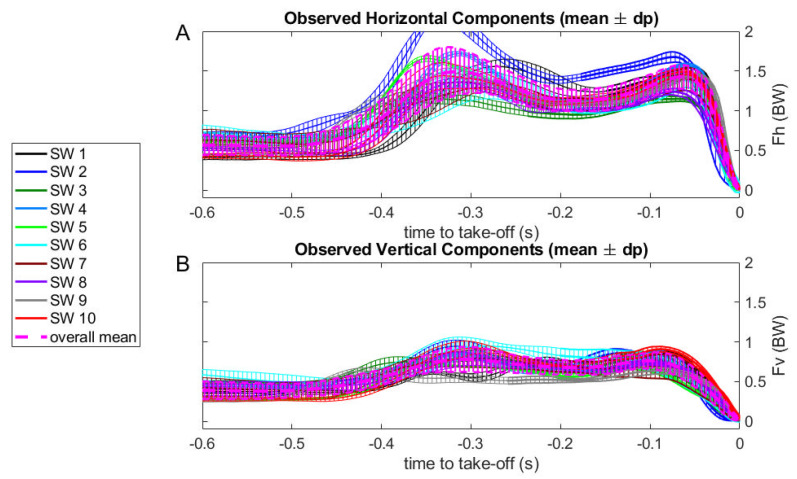
Mean horizontal ((**A**) panel) and vertical ((**B**) panel) force–time curves and respective standard deviations for each swimmer (SW) and overall sample trials expressed as time to take-off for the backstroke start technique.

Following the starting signal, the backstroke swimmers’ initial movements occur between 0.5 and 0.4 s before the take-off instant (Figure 8A,B), and the hands-off instant varies among swimmers, occurring between 0.25 and 0.15 s before the take-off (for each swimmer curve, the line thickness is increased at hands-off, Figure 8A,B). Most of the swimmers showed consistent force–time curves, evidencing high stability, with standard deviations between 0.02 and 0.06 BW, with exception of swimmers six and seven (with 0.1 BW; Figure 8A,B). Considering the swimmers 40 force–time curves all together, they showed a standard deviation of 0.14 BW and 0.1 BW for horizontal (Figure 8A) and vertical (Figure 8B) components, respectively.

In conclusion, each one of the swimmers in the sample has a consistent way of force production at the swimming start, although different swimmers use different strategies. Since our model only applies after hands-off, in the remaining figures we will only show the force curves after this event and until take-off.

**Figure 9:**
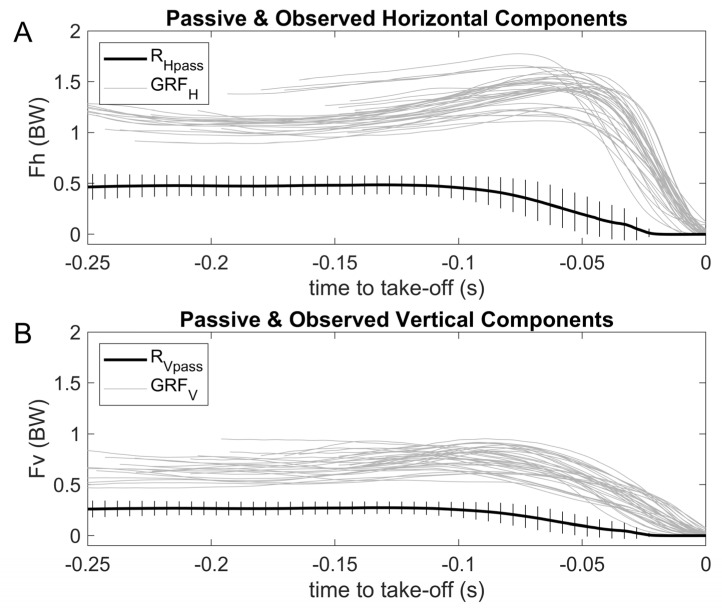
Mean (plus SD) passive curve for all swimmers (continuous black line) and the 40 observed swimmers’ backstroke-start trials (continuous grey lines) $GRF_{H}\left(t\right)$ and $GRF_{V}\left(t\right)$ curves ((**A**) and (**B**) panels, respectively). Passive and observed forces were synchronized to take-off.

**Figure 10:**
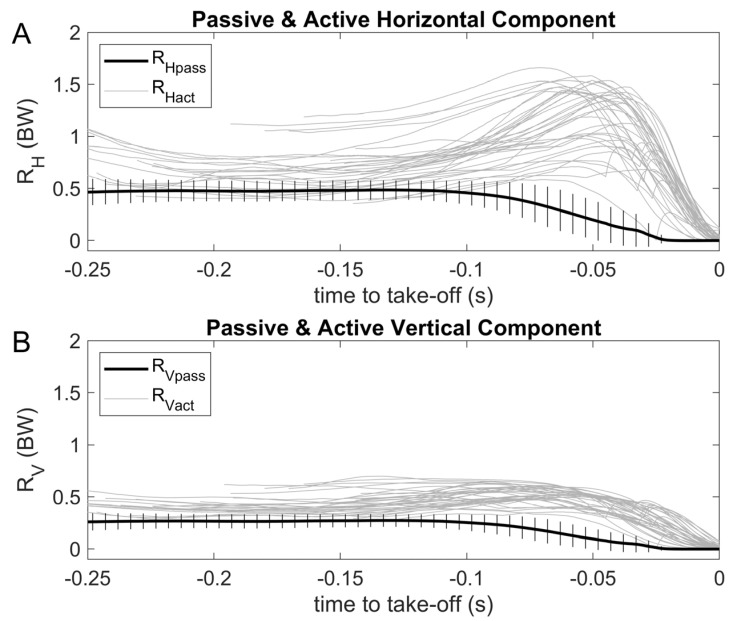
Mean (plus SD) passive curve for all swimmers (continuous black line) and active swimmers (continuous grey lines) $R_{\mathrm{Hpass}}\left(t\right)$ and $R_{\mathrm{Hact}}\left(t\right)$, and $R_{\mathrm{Vpass}}\left(t\right)$ and $R_{\mathrm{Vact}}\left(t\right)$ curves ((**A**) and (**B**) panels, respectively). Passive and active forces were synchronized to take-off.

Rigid body fall $R_{\mathrm{Hpass}}\left(t\right)$ and $R_{\mathrm{Vpass}}\left(t\right)$ curves showed stability from the hands-off until 0.12 s before the take-off, followed by a progressive descending profile (Figure 9). In opposition to this, most of swimmers seem to evidence within the trials a slightly increasing $GRF_{H}\left(t\right)$ and $GRF_{V}\left(t\right)$ profile from hands-off until reaching a peak value just before take-off. $GRF_{H}\left(t\right)$ had the most outstanding magnitude, with peak values occurring later than those of $GRF_{V}\left(t\right)$ (~0.04 vs 0.08 s before take-off, respectively; Figure 9). Figure 10 shows the same $R_{\mathrm{Hpass}}\left(t\right)$ and $R_{\mathrm{Vpass}}\left(t\right)$ curves as Figure 9, displaying a stable curve between 0.25–0.12 s before take-off, which was followed by a descending profile, concomitant with progressive increases in $R_{\mathrm{Hact}}\left(t\right)$ and $R_{\mathrm{Vact}}\left(t\right)$. Since the splitting of the observed force curves into passive and active components is obtained through a *θ*(*t*)-mapping and not a *t*-mapping, some artifacts may appear in the curves (Figure 10).

## 4. Discussion

Swimming-start $GRF_{H}\left(t\right)$ and $GRF_{V}\left(t\right)$ evaluations have here highlighted swimmers’ strategies for achieving a proper take-off angle to attain a longer flight distance. Since this is an important starting skill [1,3,6], accurate tools were designed to assess and interpret the generation of the swimming-starts’ external force for research purposes and training feedback [1,12,20]. The current study used a previously developed algorithm [6] to interpret the mechanisms that are responsible for the backstroke start $GRF_{H}\left(t\right)$ and $GRF_{V}\left(t\right)$ generation (as based in fundamental mechanics). From hands-off until ~0.12 s before take-off, $R_{\mathrm{Hpass}}\left(t\right)$ and $R_{\mathrm{Vpass}}\left(t\right)$ depicted a stable profile, followed by a gradual reduction concurrent with rises in $R_{\mathrm{Hact}}\left(t\right)$ and $R_{\mathrm{Vact}}\left(t\right)$ [12], showing that even in the absence of any active propulsion effort, real propulsion can be observed [6]. Therefore, it was noticed that two start trials from the same swimmer would produce identical $\vec{GRF}\left(t\right)$ signature patterns, with eventual dissimilarities occurring due to different active and passive force contributions. Consequently, the analysis of these curves would enable more objective and accurate feedback [5,16,21], a need that would become even greater when realizing that the differences between elite athletes have decreased considerably over the years [16,22].

Swimmers’ inertia changed due to the different (most-tucked or most-extended) backstroke-start inter-segmental positions that were used in the computer simulations. This allowed for the calculation of $\theta \left(t\right)$ as a crucial parameter in the CM–COP direction and the separation of ${\vec{R}}_{\mathrm{Passive}}\left(t\right)$ and ${\vec{R}}_{\mathrm{Active}}\left(t\right)$ components. Contrarily to the ventral start technique [6], the $I_{ZZ}$ value almost tripled in the most-tucked vs the most-extended body positions, in line with the complexity of the backstroke-start performance [7,9,23]. In the backstroke start, swimmers had quickly to change from a tucked positioning (mostly sustained by the upper limbs even when using the wedge) to an effective segmental organization to generate proper lower-limb propulsion [7,9,11]. Small $I_{ZZ}$ values enabled higher angular accelerations around the halluces and evidenced that the beginning of the movement was clearly a rotation [7,9,11], while the last contact moments showed more propulsion out of the wall effort. Contrarily to the previous studies, the present findings showed a detailed technical analysis based on the horizontal and vertical force–time curve profiles generated by muscular-based biomechanical actions and body weight dynamical effects during a commonly used backstroke start variant. The absolute force and impulse assessments are valuable performance indicators [8,10]; however, the understanding of the steering strategy adopted considering the relative position and orientation of body segments as they changed during the backstroke start might help coaches and swimmers to perform the task in a more effective way.

Swimmers who performed the symmetric $GRF_{H}\left(t\right)$ and $GRF_{V}\left(t\right)$ ventral grab start propelled themselves out of the block at a ~45° take-off angle [6] and $GRF_{H}\left(t\right)$ and $GRF_{V}\left(t\right)$ at the kick start had a similar magnitude profile after the rear-foot take-off [1,14,24]. However, these variables have not shown a similar pattern in the backstroke start, even with the wedge [8,12], probably due to a smaller $\theta \left(t\right)$ angle constraint. The take-off angle is a backstroke-start performance determinant [7,9,10], and is critical for the flight phase extent and duration [9,11]. The backstroke starts with vs. without wedge showed a greater take-off angle [11] because of friction mechanism obviation [8,12]. Elite swimmers used a larger hip joint angular velocity to achieve proper steering postures on the take-off and entry angles [7,14], and the ${\vec{R}}_{\mathrm{Passive}}\left(t\right)$ contribution after hands-off, followed by the ${\vec{R}}_{\mathrm{Active}}\left(t\right)$ generation, highlighted that major postural adjustments for proper take-off angle should be performed until the reduction of meaningful inertial effects in order to optimise backstroke-start performance determinants before and after the hands-off instant [7,12].

The current study’s $\theta \left(t\right)$ angle had a sharp value reduction ~0.1 s before the take-off, in agreement with progressive increases in $GRF_{H}\left(t\right)$ and $GRF_{V}\left(t\right)$. Following the hands-off, swimmers continued rotating the trunk and upper limbs, which contributed to the appearance of a maximum $GRF_{V}\left(t\right)$ before the $GRF_{H}\left(t\right)$ peak [13]. $GRF_{V}\left(t\right)$ peak values had already been noticed when using the wedge [10] and were not so pronounced as $GRF_{H}\left(t\right),$ due to essential backward steering before the take-off [8,11]. Notwithstanding this, $GRF_{V}\left(t\right)$ was indicated as a crucial factor for successful ventral swimming-starts performance [1,5,15], and a larger $GRF_{H}\left(t\right)$ peak just before take-off is a cardinal result for backstroke-start propulsion [12,13,24]. Even though the main contribution of $GRF_{V}\left(t\right)$ during the backstroke start with the wedge is expected to be denoted before the hands-off, no kinetic effect was observed in backstroke start kinetics with the wedge [8]. The present study reinforces the fact of backstroke-start performance complexity to generate the vertical component for propulsion (cf. previous findings [8]), but also evidences from the active and passive force–time curve analysis the optimal time for segmental rotations and effective propelling force. Moreover, contrarily to the passive force, the active component is apparently more susceptible to high variability (cf. [6]), which suggests that the selection of the proper backstroke-start technical variation should be mainly based on optimised propulsive muscular actions preceding the take-off.

The $\theta \left(t\right)$-mapping methodology developed and implemented before on the $\vec{GRF}\left(t\right)$ grab-start technique to split ${\vec{R}}_{\mathrm{Passive}}\left(t\right)$ and ${\vec{R}}_{\mathrm{Active}}\left(t\right)$ components [6] was used in the current study to characterise the backstroke start. It exhibited an evident $R_{\mathrm{Hpass}}\left(t\right)$ and $R_{\mathrm{Vpass}}\left(t\right)$ contribution from ~0.2–0.12 s before the take-off (progressively decreasing), in opposition to the grab-start profile [6]. The grab-start conditions are different, considering the $\theta \left(t\right)$ angle, with swimmers undergoing ${\vec{R}}_{\mathrm{Passive}}\left(t\right)$ effects during a longer period and, consequently, revealing a greater $R_{\mathrm{Vpass}}\left(t\right)$ contribution. In the grab-start technique, swimmers evidenced $R_{\mathrm{Hact}}\left(t\right)$ and $R_{\mathrm{Vact}}\left(t\right)$ just after the hands-off, which means that over 60% of the block time has been influenced by inertial components. Fine control and timing of force production is necessary to find the optimal flight phase trajectory and at the same time prepare for a smooth water entry with the least possible resistance [22]. The main task for the swimmer has environmental and organic constraints that are faced during the most propulsive starting wall instants, and subtle differences may distinguish swimmers and individual swimmers’ trials as a consequence of environmental changes, training procedures, or learning phenomena [6,7,8]. In fact, between-participant kinetic variability was previously noticed when the backstroke start was performed with the wedge, which was explained by differences in the swimmers’ technique or strength [8]. In opposition to ${\vec{R}}_{\mathrm{Active}}\left(t\right)$, ${\vec{R}}_{\mathrm{Passive}}\left(t\right)$ is dependent on swimmer’s structure and inertial components, diminishing the degrees of freedom involved in the swimming start movement and the consequent variability.

Taking into consideration the backstroke start, the ${\vec{R}}_{\mathrm{Passive}}\left(t\right)$ influence revealed a more discreet profile for $R_{\mathrm{Hpass}}\left(t\right)$ and $R_{\mathrm{Vpass}}\left(t\right)$ components, which were substantially replaced by $R_{\mathrm{Hact}}\left(t\right)$ and $R_{\mathrm{Vact}}\left(t\right)$ ~0.12 s before take-off. The upper and lower limbs’ high propulsive contribution during starts has been evidenced since the 1980s [10,12], leading researchers to propose diverse resistance-training programs to improve strength and power [3,5]. However, studies regarding the effects of force generated by swimmers on their starting performances are very scarce. The display of the configuration of swimmers’ body segments when generating ${\vec{R}}_{\mathrm{Passive}}\left(t\right)$ and ${\vec{R}}_{\mathrm{Active}}\left(t\right)$ force profiles, as well as $\theta \left(t\right)$ data, might benefit coaches and sports scientists when designing backstroke-start strength tests, implementing strength training, and selecting the most proper backstroke-start variant for better performance [3,5] (with a combined kinetics and kinematics analysis; e.g., [4,12,13]).

Notwithstanding the current study’s originality and relevance, limitations and further research directions should be addressed. The previously developed algorithm that allows the separation of ${\vec{R}}_{\mathrm{Passive}}\left(t\right)$ from ${\vec{R}}_{\mathrm{Active}}\left(t\right)$ components in the grab start can be applied in the backstroke start from hands-off onwards. However, improvement of the algorithm’s capability to assess $\vec{GRF}\left(t\right)$ components in view of the COP translation from the starting beep to the hands-off should be taken into consideration in future work, since the defined start variant might exhibit swimmers’ setup adaptations, which might affect kinematic and kinetics data (e.g., [8,10]). A swimmer’s mass is directly connected to anthropometric characteristics like volume, volumetric mass, CM position, height, and arm span, with the current model revealing small sensitivity to it [6]. However, the impact of different swimmers’ levels of mass, $I_{zz}$, and CM–COP values should be analysed, since more-proficient swimmers might produce better segment positioning and muscular effective actions [3,7,10].

## 5. Conclusions

The current study revealed that the backstroke start is performed with the generation of ${\vec{R}}_{\mathrm{Passive}}\left(t\right)$ and ${\vec{R}}_{\mathrm{Active}}\left(t\right)$ forces and, even considering wedge use, it is considered a complex motor task. Starts performed in-water imply a small $R_{\mathrm{Hpass}}\left(t\right)$ and $R_{\mathrm{Vpass}}\left(t\right)$ contribution during a short period just after the hands-off instant. ${\vec{R}}_{\mathrm{Passive}}\left(t\right)$ influence was sharply replaced by $R_{\mathrm{Hact}}\left(t\right)$ and $R_{\mathrm{Vact}}\left(t\right)$ components, pointing out that swimmers should focus on trunk and upper-limb rotations for proper steering position from the beep until the post-hands-off instants. Following this critical instant, effective muscular actions are essential to potentiate horizontal propulsion before take-off. These findings might help coaches to improve resistance-training programs’ accuracy and select the proper technical variant of start while considering swimmers’ body segments at the ${\vec{R}}_{\mathrm{Active}}\left(t\right)$ instants as major contributors for propulsion and, consequently, enhancing the overall backstroke-start performance.

## Figures and Tables

**Table 1:** Inertia tensors expressed in $\left(\mathrm{kg}\cdot m^{2}\right)$ calculated for hallux rotation axis in the most-tucked and the most-extended rigid articulated body positions.

Rigid Articulated Body Positions	Moment of Inertia Matrix Components
Most-tucked	$\left[\begin{array}{ccc} I_{XX} & I_{XY} & I_{XZ}\\ I_{YX} & I_{YY} & I_{YZ}\\ I_{ZX} & I_{ZY} & I_{ZZ} \end{array}\right]=\left[\begin{array}{ccc} +37.0068 & -16.0489 & -0.0006\\ -16.0498 & +11.2819 & +0.0002\\ -0.0006 & +0.0002 & +46.5009 \end{array}\right]$
Most-extended	$\left[\begin{array}{ccc} I_{XX} & I_{XY} & I_{XZ}\\ I_{YX} & I_{YY} & I_{YZ}\\ I_{ZX} & I_{ZY} & I_{ZZ} \end{array}\right]=\left[\begin{array}{ccc} +109.9163 & -44.298 & -0.0009\\ -44.298 & +22.5456 & +0.001\\ -0.0009 & +0.001 & +130.6885 \end{array}\right]$

## Data Availability

Data will be available under reasonable request.
